# Impact of transfusion on patients with sepsis admitted in intensive care unit: a systematic review and meta-analysis

**DOI:** 10.1186/s13613-016-0226-5

**Published:** 2017-01-04

**Authors:** Claire Dupuis, Romain Sonneville, Christophe Adrie, Antoine Gros, Michael Darmon, Lila Bouadma, Jean-François Timsit

**Affiliations:** 1 UMR 1137 - IAME Team 5 – DeSCID: Decision Sciences in Infectious Diseases, Control and Care Inserm/Univ Paris Diderot, Sorbonne Paris Cité, 75018 Paris, France; 2 Medical and Infectious Intensive Care Unit, AP-HP, Hôpital Bichat Claude Bernard, Paris Diderot University, 75018 Paris, France; 3 Medical-Surgical ICU, Delafontaine Hospital, 2 rue du docteur Delafontaine, BP 279, 93 205 Saint-Denis, France; 4 Medical-Surgical ICU, Versailles Hospital, 177 Rue de Versailles, 78150 Le Chesnay, France; 5 Medical ICU, Saint-Etienne University Hospital, Avenue Albert Raymond, 42270 Saint-Priest-en-Jarez, France

## Abstract

Red blood cell transfusion (RBCT) threshold in patients with sepsis remains a matter of controversy. A threshold of 7 g/dL for stabilized patients with sepsis is commonly proposed, although debated. The aim of the study was to compare the benefit and harm of restrictive versus liberal RBCT strategies in order to guide physicians on RBCT strategies in patients with severe sepsis or septic shock. Four outcomes were assessed: death, nosocomial infection (NI), acute lung injury (ALI) and acute kidney injury (AKI). Studies assessing RBCT strategies or RBCT impact on outcome and including intensive care unit (ICU) patients with sepsis were assessed. Two systematic reviews were achieved: first for the randomized controlled studies (RCTs) and second for the observational studies. MEDLINE, EMBASE, Web of Science Core Collection, Cochrane Central Register of Controlled Trials, Cochrane Database of Systematic Reviews and Clinical Trials.gov were analyzed up to March 01, 2015. Der Simonian and Laird random-effects models were used to report pooled odds ratios (ORs). Subgroup analyses and meta-regressions were performed to explore studies heterogeneity. One RCT was finally included. The restrictive RBCT strategy was not associated with harm or benefit compared to liberal strategy. Twelve cohort studies were included, of which nine focused on mortality rate. RBCT was not associated with increased mortality rate (overall pooled OR was 1.10 [0.75, 1.60]; *I* ^2^ = 57%, *p* = 0.03), but was associated with the occurrence of NI (2 studies: pooled OR 1.25 [1.04–1.50]; *I* ^2^ = 0%, *p* = 0.97), the occurrence of ALI (1 study: OR 2.75 [1.22–6.37]; *p* = 0.016) and the occurrence of AKI (1 study: OR 5.22 [2.1–15.8]; *p* = 0.001). Because there was only one RCT, the final meta-analyses were only based on the cohort studies. As a result, the safety of a RBCT restrictive strategy was confirmed, although only one study specifically focused on ICU patients with sepsis. Then, RBCT was not associated with increased mortality rate, but was associated with increased in occurrence of NI, ALI and AKI. Nevertheless, the data on RBCT in patients with sepsis are sparse and the high heterogeneity between studies prevents from drawing any definitive conclusions.

**Electronic supplementary material:**

The online version of this article (doi:10.1186/s13613-016-0226-5) contains supplementary material, which is available to authorized users.

## Background

Anemia is frequent in intensive care medicine and is associated with increased morbidity and worsened outcome [1]. In severe sepsis and septic shock patients, the red blood cell transfusion (RBCT) threshold remains controversial. Indeed, RBCT may improve oxygen delivery into tissues and is one of the main interventions of the early goal-directed therapy (EGDT) [2]. Consequently, for many years, a 10 g/dL hemoglobin level has been recommended during the early phase of septic shock. Nonetheless, adverse events such as overload, infectious complications and immunomodulatory effects of transfusion have been described [3–5]. Therefore, lowering the transfusion threshold might reduce the volume of transfusion in patients and may prove beneficial [6]. Indeed, a restrictive transfusion strategy has been recommended and acknowledged for most of the non-septic patients but is still not fully established for ICU patients with sepsis [7, 8].

As a result, two main questions remain open regarding septic patients: first, what is the optimal transfusion threshold, and second, does transfusion impact on clinically relevant outcomes, including mortality, and occurrence and/or duration of organ failure. In that context, several studies specifically focused on septic ICU patients were recently conducted. For instance, a randomized controlled trial (RCT) published in 2014 [9] showed that lowering the transfusion threshold was not harmful for patients with septic shock. Then, several observational studies have been recently published with controversial results regarding the impact of transfusion on mortality [10–13]. Until now, no systematic review has specifically focused on the impact of transfusion neither on the outcome nor on the transfusion thresholds for septic ICU patients. In this context, the purpose of this study was to perform a systematic review and meta-analysis regarding transfusion among critically ill adults with sepsis in order to address these two questions: (1) what are the benefits or harms of restrictive transfusion strategies compared to liberal ones, and (2) what is the impact of transfusion on septic critically ill patients with regard to their mortality, the occurrence of nosocomial infection or other organ failures.

## Methodology

### Data sources and searches

This review was systematic and comprised two subcategories: (1) RCTs dealing with the comparison of two transfusion thresholds; and (2) observational studies on the impact of transfusion on the outcome among ICU patients with sepsis. The preferred reporting items for systematic reviews and meta-analysis (PRISMA) guidelines statement [14] was followed for the randomized controlled trials, and the meta-analysis of observational studies in epidemiology (MOOSE) guidelines [15] applied for the observational studies.

The research question was formulated according to the participants, interventions, comparisons and outcomes (PICO) model as follows: P, septic ICU adults; I, red blood cell transfusion for the observational studies and restrictive strategy for the RCTs; C, no transfusion for the observational studies and liberal strategy for the RCTs and O, all the outcomes were considered, such as death, nosocomial infection, occurrence of acute lung injury, except outcomes (events) collected during the first week after ICU admission. As a result, we only selected studies which included ICU patients with severe sepsis or septic shock, and that assessed the effects of RBCT on outcome for observational studies and that compared two transfusion thresholds (restricted versus liberal strategies) for the RCTs. Relative risk (RR), odds ratio (OR), hazard ratio (HR) or standardized incidence/mortality ratio (SIR/SMR) with their specific 95% confidence intervals (CIs) had to be reported or were calculated from the article. We only focused on English- and French-written articles published between the January 01, 1995, and the December 31, 2014. The main search was performed in November 2014 and updated in March 2015. We did not consider studies in some specific populations: pediatric patients, trauma patients, patients with burns, patients undergoing surgery and among them cardiac surgery, and patients with acute coronary syndromes or with acute brain injury. We also excluded studies that assessed the benefice of a systematic leukoreduction versus a non-leukoreduction of the packed RBC, and studies evaluating the effects of red cell storage duration. Research sources included MEDLINE, central, Web of Science Core Collection, Cochrane Central Register of Controlled Trials, Cochrane Database of Systematic Reviews and ClinicalTrials.gov. Search terms were “sepsis,” “septic shock,” “transfusion,” “intensive care,” “threshold,” “cohort,” “randomized trials,” “outcome.” Boolean algorithms with specific terms were elaborated for each of the two subcategories of studies. They are listed in Additional file 1.

### Study selection

Study selection was conducted through independent review. Two independent reviewers (C.D. and J.F.T.) examined abstracts for eligibility. In case of disagreement based on abstract, the full-text article was obtained to determine the study eligibility. All duplicated studies or studies that only described methods of the trials without reporting results or studies with ineligible comparison or inadequate outcome were excluded.

### Data extraction and quality assessment

A data extraction form was developed prior to articles review, enabling to record the first author’s last name, publication year, period of inclusion, country, study design, inclusion criteria, number of participating sites, number of participating patients, proportion of patients with sepsis, probability of death at admission extrapolated from the severity score at ICU admission, statistical methods and covariates used for adjustment, patients’ outcome(s) and risk estimates with their 95% confidence intervals. From the randomized trials, we collected hemoglobin thresholds, number of participating patients and main results. Data extraction was conducted independently by two investigators, with subsequent discussion and resolution of discrepancies by consensus. In case of missing data, we contacted the authors of the original studies.

### Meta-analysis

Effects estimates were primarily presented as adjusted odds ratios (ORs). Unadjusted ORs were used in the absence of adjusted OR. Because of the high prevalence of the events, OR could not be approximated by the hazard ratios (HRs) [16]. As a result, if the studies only reported HR, data were used to calculate non-adjusted odds ratios that were used into the meta-analysis. We pooled individual study data using Der Simonian–Laird proportion methods. Due to anticipated heterogeneity, we used a random-effects meta-analysis, which considered both within-study and between-study variations. Heterogeneity was assessed using *I* ^2^ statistics, Chi-square test, Tau^2^ and by visualization in a funnel plot. For the Q statistic, a *p* value of less than 0.10 was used as an indication of the presence of heterogeneity; for *I* ^2^, a value >50% was considered a measure of severe heterogeneity. To explore potential heterogeneity between studies, several subgroup analyses were conducted, first with statistical strategies and then in accordance with the different outcomes. Then, meta-regressions were performed with the first year of inclusions and the estimated patients’ severity of illness at admission as covariates. Sensitivity analyses were conducted based on extraction of potential outliers.

Publication bias was evaluated using a funnel plot of a trial’s effect size against the standard error. All statistical analyses were performed using RevMAn, version 5.3 (Nordic Cochrane Centre, Cochrane Collaboration, Copenhagen, Denmark), except for the meta-regression which was performed with the package metaphor with the “R” software (version 2.13.0 R Foundation for Statistical Computing, Vienna, Austria). A two-tailed *p* value of less than 0.05 was considered to be statistically significant.

### Quality assessment and risk of bias across the studies

To assess the quality of the randomized controlled studies, the risk of bias tool was used [17]. As far as observational studies are concerned, most of the quality scoring remained controversial with lack of validity. Nevertheless, we decided to use a modified version of the Newcastle–Ottawa quality assessment scale [18]. The item concerning control group was not taken into account because all controls and cases were extracted from the same database. In the case of prospective digital cohort, the ascertainment of cohort and patients’ outcomes could be considered of high quality and thus associated with a low risk of bias. On the opposite, a high risk of bias was attributed to retrospective data collection from manual records or administrative databases. The follow-up periods of all studies were long enough. If more than 20 percent of the data were missing, we considered the risk of bias as high.

## Results

### Search results

Two main analyses were thus conducted. The first one focused on randomized controlled trials comparing restrictive versus liberal transfusion strategies and the second one on cohort studies.

### Description of the RCT

**Fig. 1:**
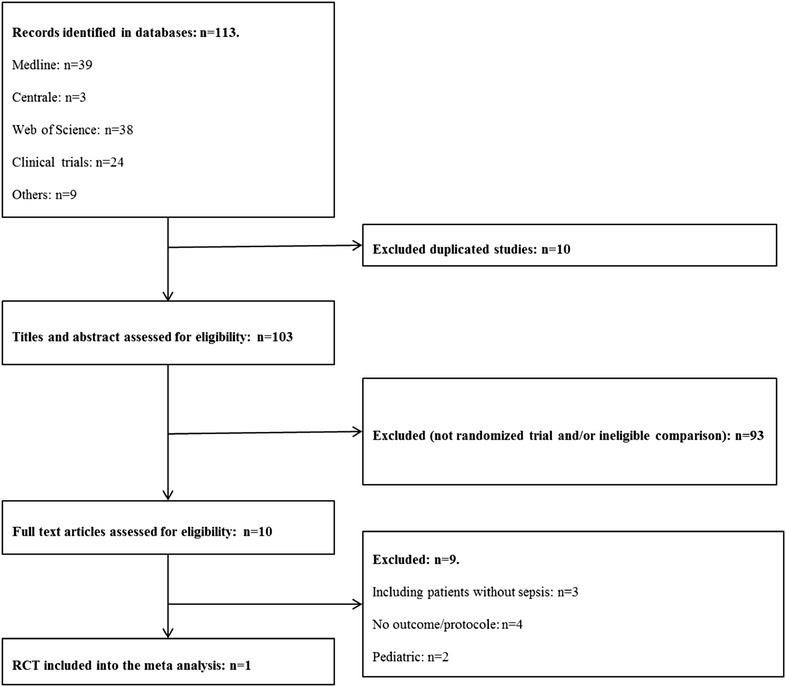
Flow diagram of search strategy for the randomized controlled trials (RCTs)

One hundred and thirteen articles were identified after consultation of the different databases. Among them, 103 were assessed based on title and abstract and 10 assessed on full-text articles. Finally, only one study was included, conducted by Holst [9] (Fig. 1). The solely eligible study concluded that none of the strategies was associated with an increased 90-day mortality (RR 0.94 [0.78–1.09], *p* = 0.4). In the restrictive strategy group, patients were less transfused [unit transfused during ICU stay: 1 [0–3] versus 4 [2–7], and proportion of patients free of transfusion during ICU stay: 176 (36.1%) vs. 6 (1.2%)]. However, protocol violations were more frequent in the restrictive group than in the liberal one (5.9 vs. 2.2%, *p* = 0.004) because of ischemic events or acute bleeding most of the time. Summaries of this study and its quality assessment are reported in Table 1 and in Additional file 2: Tables S1 and S2. Of note, in a post hoc analysis, a meta-analysis of the randomized controlled trials of ICU patients that included septic and non-septic patients and compared restrictive versus liberal strategies (3 studies), was achieved and did not reveal differences in the risk of mortality (RR 0.81 [0.63–1.04] [*I* ^2^ = 41%, *p* = 0.18)] (Additional file 2: Figure S2) [6, 9, 19].

**Table 1:** Characteristics of the included studies

References	Country	No. of site	Data collection	No. of patient	% med/chir	Primary outcome	Inclusion criteria	Inclusion period	Severity score^a^ Mean (sd) or median [IQ]	Number of RBC transfused (mean (sd) or median [IQR])	Mean Hb level before transfusion	Statistical model	Leukoreduction	% of death
*Randomized controlled studies*														
Holst [9]	Denmark	32		998				2011–2013	SII 51				100%	90-day mortality rate: 43% versus 45%, *p* = 0.44
*Cohort studies*														
Micek [21]	USA	1	Prospective	102	60/40	H death	ss and dotrecogin	2002–2004	AII S: 26.3 (5.1) No S: 29.7 (6.1)	S: 1.7 (2.5) No S: 1.3 (2.7)	–	LR	–	42.2
Fuller [52]	USA	1	Retrospective	93	From ED > 95%	H death	ss	2005–2008	AII T: 21.1 No T: 20.3	4.56	–	UNI	–	N (%) (transfused/not transfused) H death: 14 (41.2%); 20 (33.9%); *p* < 0.05
Parsons [12]	USA	20	Prospective	285	90/10	D28 death	S and ALI	2000–2005	AIII T: 118 (27) No T: 103 (2)	–	Baseline (Hb)^b^ T: 8.5 (1.4) No T: 9.7 (1.4)	LR	–	D28: transfused: 10 (50%), no transfused: 19 (29%)
Perner [10]	Denmark	5	Prospective	164	–	D28 death	ss	2009–2009	SII 54 (46–67)	507 ui of RBCs^c^ [900 ml (490–1405)]	Baseline (Hb) T: 8.58 [7.36–9.28] No T: 9.76 [8.64–10.72]	LR	100%	D30: 40%
Park [13]	Korea	12	Prospective	1054	100% from ED	D28 death	ss or ss (63.2%)	2005–2009	AII No T: 17.4 (7.1) T: 21.2 (7.4)	–	Before transfusion 7.7 (1.2)	COX + PS	15%	T versus no T D28: 24.3% versus 38.8%; *p* = 0.007
Rosland [11]	Denmark	10	Prospective	213	58/42	D90 death	ss	2013	SII 51 (40–65)	mL (median[IQ]) D1: 490 (300–735); D2: 490 (245–490); D3: 245 (245–490); D4:245 (245–245)	D1 (Hb) T: 7.3 (6.8–7.9) No T: 10.0 (8.9–11.1)	LR	100%	28-day mortality rate, all 102 (48)
Sadaka [53]	USA	1	Prospective	396	–	H death	ss (60%)	2011–2013	SOFA T: 8.6 (3.9) Np T:8.4 (3.4)	–	–	LR + matching	–	
Na [54]	Asia	8	Prospective	556	100% from ED	H death	ss or ss (67%)	2008–2009	AII 22 (16.27)	–	Baseline (Hb) All 11.6 [9.7, 13.2]	LR	–	In-hospital mortality (%) 166 (29.9)
Juffermans [24]	Netherlands	Multi	Retrospective	134	–	NI	s	2004–2007	AII Non T:19 [14–38] T: 24 [145–42]	No infection: 2 [2–5] Infection: 5.5 [2–7]	–	LR	100%	
Erbay [25]	USA	1	Retrospective	73	ICU/Burn unit (%) 47/26	NI	ICU and KT infection	1998–2002	–	–	–	COX	–	
Iscimen [26]	Turkey	1	Prospective	162		ALI	ss without ALI	2004–2007	AIII ALI:61 [49–72] No ALI:55 [44–64]	–	–	LR	–	ICU death, n (%)ALI:27 (38),no ALI 10 (11), *p* = 0.001
Plataki [27]	USA	1	Prospective	390		RIFFLE	ss	2005–2007	AIII No AKI: 77 [63–96] AKI: 92 [72–109]	–	Baseline (Hte) AKI: 32 [29–38] No AKI: 31 [27–36]	LR	–	In-hospital mortality rate, n (%) no AKI: 52 (34)versus AKI: 115 (49);*p* = 0.005

H, hospital; H death, in-hospital mortality rate; D, day; D-90 death, 90-day mortality rate; ICU, intensive care unit; NI, nosocomial infection; KT, catheter; MV, mechanical ventilation; ss, severe sepsis; ARDS, acute respiratory distress syndrome; Hrs, hours; ALI, acute lung injury; AII = APACHE II; S^λ^, SAPSS; SII, SAPS II; AIII = APACHE III, SOFA, Sequential Organ Failure Assessment; RBC, red blood cell; PRBC, packed red blood cell; T, transfused; BSI, blood stream infection; VAP, ventilatory-associated pneumonia; AKI, acute lung injury; Ui, unit; LR, logistic regression; UNI, univariate; PS, propensity score; No., number; Hb, hemoglobin; SICU, surgical ICU; RIFLE risk, injury, failure, loss of kidney function and end-stage kidney disease

^a^Severity score: values are proportions of patients unless stated otherwise; severity score is the predicted hospital death rate determined by the prognostic score on admission (APACHE 2 and APACHE 3; SAPSS 2; SOFA score)

^b^Hemoglobin levels are reported in g/dL unless stated otherwise

^c^Values are median (interquartile) unless stated otherwise

### Description of the cohort studies

**Fig. 2:**
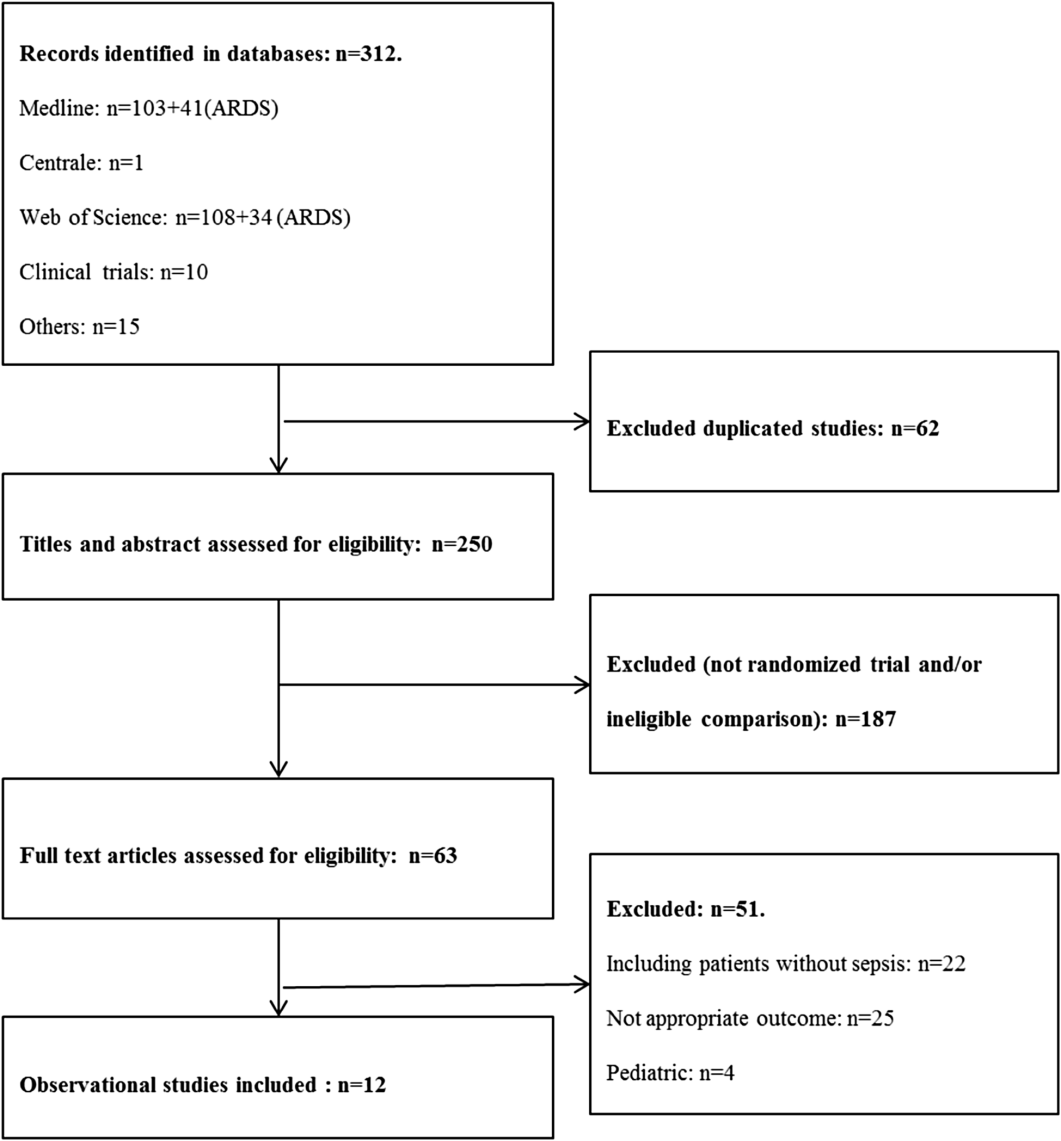
Flow diagram of search strategy for the cohort studies

Our search strategy identified 312 studies for potential inclusion. Among them, only 250 were eligible based on abstract’s assessment. Then 187 were excluded because they were not observational studies, or conducted within an inappropriate cohort (no ICU patients with sepsis), or outcome (outcomes collected during the first week after ICU admission). Among the 63 remaining studies, 12 were finally included into our systematic review (Fig. 2). In these studies, the main outcome parameter analyzed was death (8 studies), acute lung injury (1 study), acute kidney injury (1 study) and nosocomial infections (2 studies). The characteristics of the studies are summarized in Table 1 and Additional file 2: Table S3. The selected articles were actually published between 2005 and 2014, and inclusions were conducted between 1998 and 2013.

**Fig. 3:**
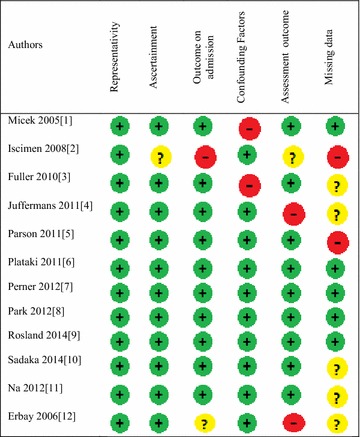
Risk of bias summary: quality assessment of the included cohort studies, using modified version of the Newcastle–Ottawa quality assessment scale

**Fig. 4:**
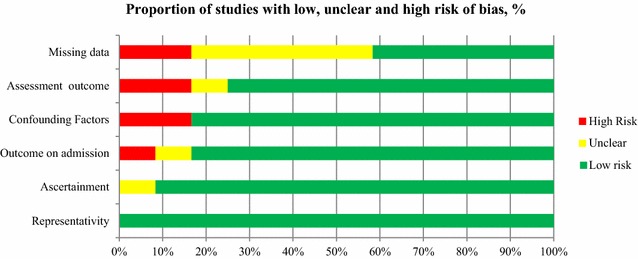
Risk of bias graph: quality assessments of included cohort studies, using modified version of the Newcastle–Ottawa quality assessment scale

With respect to the study quality assessment, the number of missing values was not reported in 55% of the studies, and no confounding factors were taken into account in around 15% of the studies. As a result, six studies could be categorized as low risk of bias. Summaries of the quality assessment are reported in Figs. 3 and 4 and in Additional file 2: Table S4 and Figure S1.

### Death

Two of the nine studies with death as outcome were excluded from the meta-analysis due to missing crude and/or adjusted odds ratios [20, 21]. As a result, 2762 patients from 7 studies were included. The median year of publication was 2012 (2012–2012). All inclusions started after 2000. Five studies started before 2010 and 2 after. Four of the studies were multicentric studies; three were prospectively conducted. The median population size was 285 [189–476], and the probability of death at admission was 42.4 [35.95–51.8]. Multivariate logistic regression models were used in five studies, Cox models in one study and univariate analyses in one study. The endpoints considered were hospital mortality (3 studies), death at day 28 (3 studies) and death at day 90 (1 study).

**Fig. 5:**
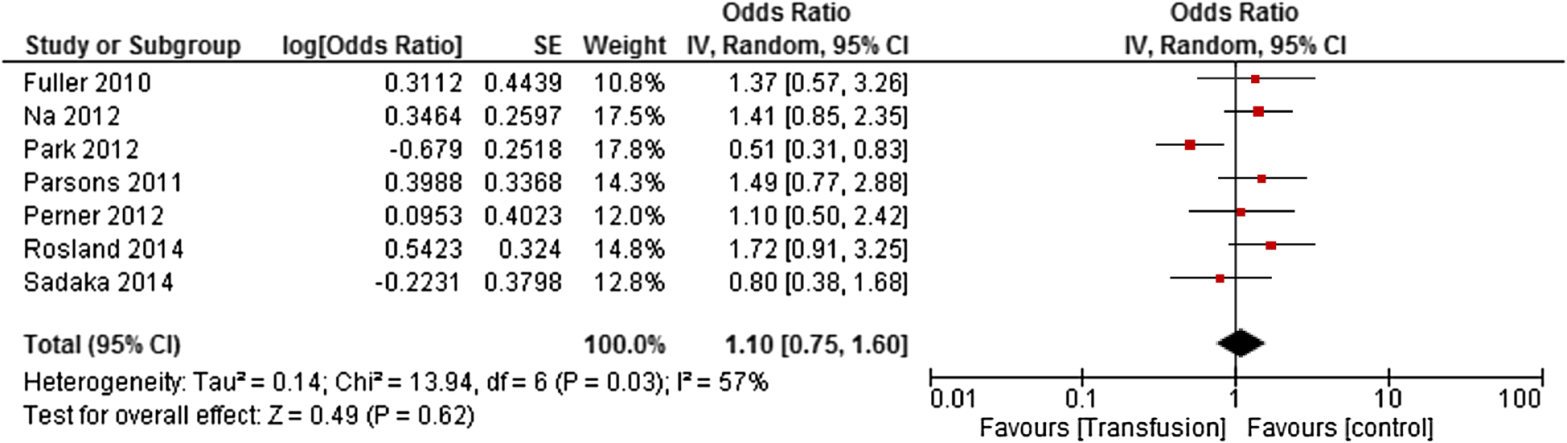
Forest plot of odds ratios: impact of red blood cell transfusions on mortality rate; *CI* confidence interval, *IV* inverse variance, *SE* standard error

**Fig. 6:**
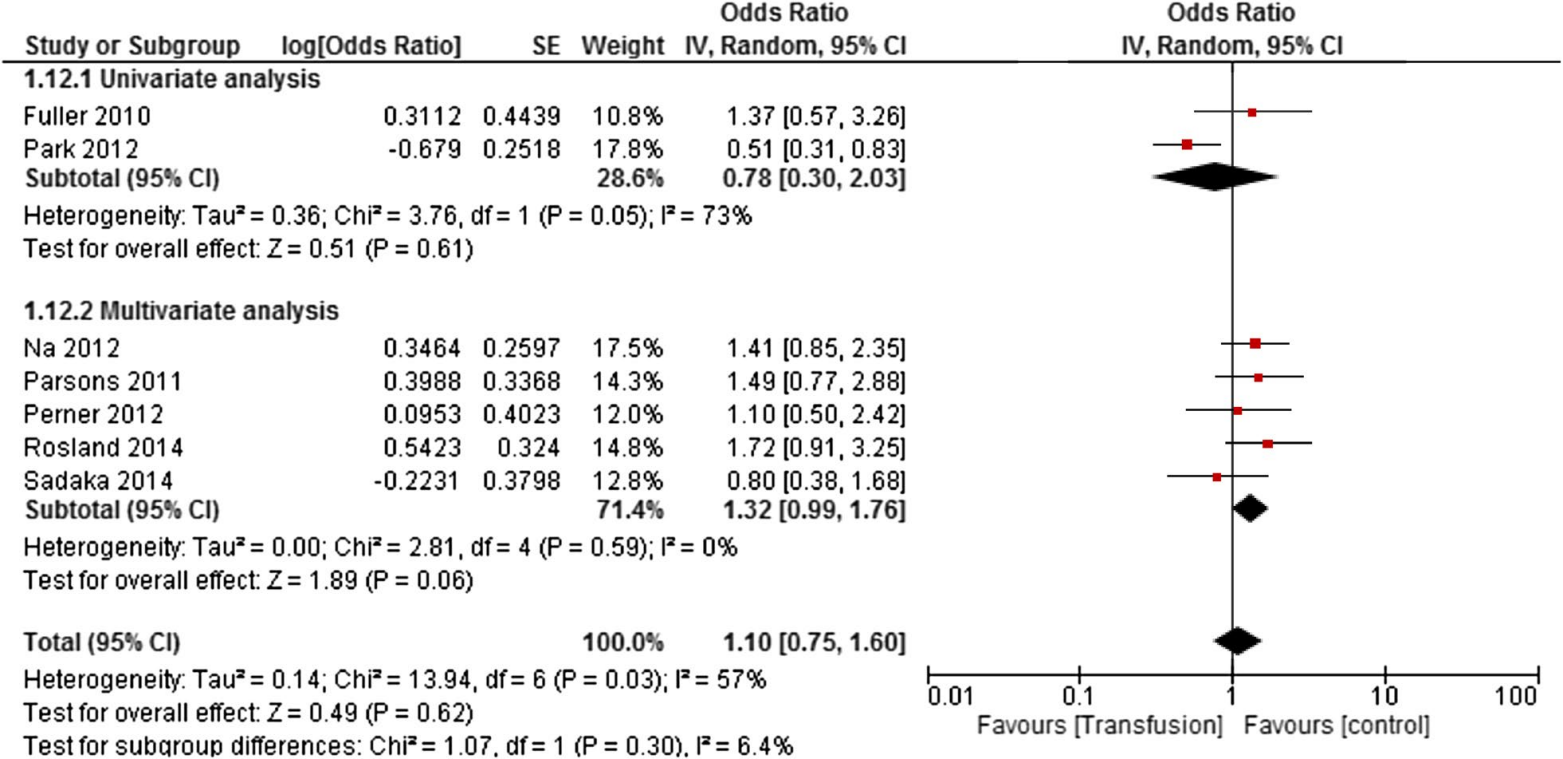
Forest plot of odds ratios: impact of red blood cell transfusions on mortality rate; subgroup analyses: statistical modeling. *CI* confidence interval, *IV* inverse variance, *SE* standard error

**Fig. 7:**
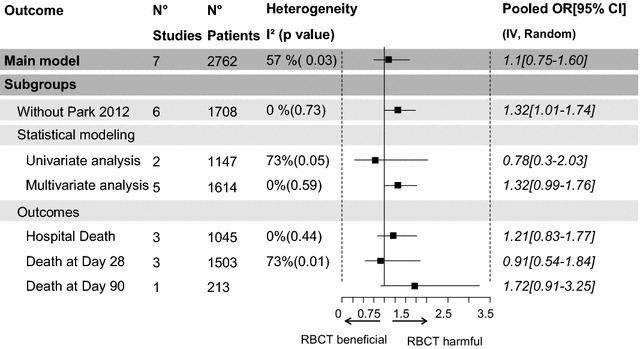
Analysis of heterogeneity: subgroup analyses: impact of red blood cell transfusion on mortality rate; *OR* odds ratio; *CI* confidence interval, *IV* inverse variance

The crude pooled odds ratio (OR) was 1.1 [0.75–1.60] (*I* ^2^ = 57%, *p* = 0.03). Among all included studies, the study of Park [13] was the only one to report a protective effect of the transfusion and was thus considered as an outlier. This study differed from the others on different points: First, it was the largest study included into our meta-analysis; second, only 62.3% of the patients included were in septic shock with the lowest probability of death at admission (27.3%); third, the hemoglobin before transfusion was one of the lowest ever reported in the literature (Hb = 7.7 g/dL). Then, it was the only study in which a Cox model on a propensity matching cohort was used. The meta-analysis was rerun without the study of Park, and the pooled OR was 1.32 [1.01–1.74] (*I* ^2^ = 0%, *p* = 0.73). Subgroups analyses and univariate meta-regressions were achieved to explore heterogeneity (Fig. 5). Then, considering the various statistical models, the pooled OR of the univariate analyses was 0.78 [0.3–2.03] (*I* ^2^ = 73%, *p* = 0.05); the one of the multivariate analyses was 1.32 [0.99–1.76] (*I* ^2^ = 0%, *p* = 0.59). Of note, the study which used a Cox model [13, 22, 23] had an HR of 0.43 [0.29–0.62]; *p* < 0.001 (Fig. 6). All the subgroup analyses and sensitivity analyses are reported in Fig. 7. Then, in the meta-regression, neither the year of the first inclusions (*I* ^2^ = 55.0%, *p* = 0.03) nor the risk of death at admission (*I* ^2^ = 55.0%, *p* = 0.03) was associated with the outcome, and none of them could explain heterogeneity. Publication bias was not obvious through the inspection of the funnel plot (data not shown).

### Nosocomial infections

Two studies dealt with nosocomial infections [24, 25]. The first one [25] started in 1998, was retrospective, monocentric, with an univariate analysis and included only 73 patients; whereas the second one [24] started in 2004, contained a multivariate analysis and included 134 patients. Their pooled OR was 1.25 [1.04–1.5] (*I* ^2^ = 0%, *p* = 0.97).

### Acute lung injury (ALI)

Only the prospective cohort of Iscimen [26] assessed the occurrence of ALI among 162 ICU patients with sepsis; it showed a deleterious effect of transfusion (OR 2.75 [1.22–6.37]; *p* = 0.016).

### Acute kidney injury (AKI)

Only the study of Plataki [27] assessed the occurrence of AKI after transfusion among ICU patients with sepsis; it demonstrated a deleterious effect of transfusion (OR 5.22 [2.1–15.8]; *p* = 0.001).

## Discussion

This systematic review suggested that a restrictive strategy could be achieved and that transfusion was not associated with increased mortality but rather with the occurrence of nosocomial infections, acute lung injuries or kidney injuries. Nevertheless, because of the limited number of studies on transfusion focusing specifically on patients with sepsis, added to the heterogeneity of the studies dealing with death, no definitive conclusions should be drawn. All those results deserved several comments.

First, the only study focusing specifically on RBCT thresholds among patients with septic shock suggested the safety of the restrictive strategy [9]. This result is in line with the results of two RCTs enrolling both septic and non-septic patients and of our post hoc meta-analysis (Additional file 2: Figure S2) [6, 9, 19]. However, some limits should be underlined. First, previously transfused patients were excluded; therefore, the safety of a restrictive strategy during the early phase of septic shock is still not explored and further study is needed. In that context, three recent RCTs (ProCESS [28], ARISE [29] and ProMISe [30]), included in a meta-analysis from Angus [31] concluded that the EGDT described by Rivers [2] was no more associated with a better outcome. Furthermore, two cohort studies showed that transfusion was safe and even beneficial during this early phase of septic shock [32, 33] and underlined that ScVO_2_ or lactate should be considered to trigger transfusion. Then, because of a partial blinding in the available RCT, many physicians did not comply with the restrictive strategy in case of risk of ischemic events. Similarly, patients with ongoing ischemic events were excluded. Consequently, the external applicability of the result could not be fully guaranteed, specifically concerning septic patients with cardiovascular events. Of note, two other studies, one after cardiac surgery [34] and another one in onco-hematology [35], were in favor of a liberal RBCT strategy. As a result, the real safety of a drastic systematic restrictive transfusion threshold among patients with sepsis must be addressed and further research is needed to determine which patients with sepsis may actually benefit from restrictive transfusion strategies. Several subgroups should be thus considered with a special interest on older patients, on patients with cardiac comorbidities, with acute ischemic event, acute brain injury or with cancer for whom a Hb threshold of 8–9 g/dL may be more appropriate.

Second, from the cohort studies, we demonstrated that transfusion did not impact death rate. This result differed from those of other studies conducted in ICU patients or after myocardial infarctions [36–42] where transfusion was deleterious. One explanation could be that patients with sepsis increased their basal metabolic and oxygen demands and thus could be more beneficial from transfusion [43]. The improvement of microcirculation by transfusion in case of baseline alteration in septic patients has also been demonstrated [44]. Nevertheless, the meta-analysis pointed out the significant heterogeneity of the transfusion effect in septic patients. The study from Park et al. [13] that showed a protective effect of transfusion was one of the main sources of heterogeneity (Fig. 7). This could be explained in part because of a lower transfusion threshold [13] (Hb = 7.7 (1.2) g/dL). Another source of heterogeneity was the statistical modeling. Indeed, some results were obtained after adjustment, and others without any, and only one study took into account time-dependent covariates by using a Cox model. Of note, the impact of deleukocytation could not be explored because of not systematically reported (Table 1).

Third, it is quite difficult to reach the real causal effect of RBCT because of treatment repetitions and of the many baseline- and time-dependent confounding factors. Until nowadays, none of the studies published have handled all those issues. Furthermore, it is important to know that Cox models with time-dependent covariates might also be biased if the proportional hazard assumption was not ascertained [45] and also because ICU discharge is an informative censor and modifies the risk of mortality and morbidity events [46]. Newer statistical causal models that can handle repetition of the treatments, such as the marginal structural models, should thus be used.

Fourth, our review suggested that transfusion was associated with the occurrence of nosocomial infection. Those results are in accordance with those of another recent meta-analysis [47], which demonstrated that restrictive strategies were associated with a reduction of the occurrence of healthcare-associated infections. Transfusion-related immune modulation (TRIM) should be considered as the main explanation [5, 48]. Mechanisms for TRIM include suppression of cytotoxic cells and monocyte activity, release of immunosuppressive prostaglandins, inhibition of interleukin-2 (IL-2) production and increase in suppressor T-cell activity and leukocytes. In this respect, the occurrence of nosocomial infections could be minimized thanks to deleukocytations of the RBCT, as demonstrated by several studies [49]. However, Jufferman also found an association between RBCT and nosocomial infections even after a systematic leukodepletion [24]. It could be explained by the few remaining leukocytes in RBCT, but also by the presence of biological active cytokines or others immunomodulating components of the red blood cells themselves. Furthermore, patients with sepsis might be more sensitive to the TRIM because of their previous immunosuppressive states.

Fifth, transfusion was associated with an increased risk of acute kidney injury. This result is based on only one study, and only few physiologic studies focused on the impact of transfusion on kidney function [44]. However, some authors believed that transfusion may elicit a renal injury similar to lung injury because of immunologic mechanisms and of overload [27, 50, 51].

Last, transfusion was associated with acute lung injury. From those studies, it was not possible to make the difference between immunologic mechanisms [transfusion-related acute lung injury (TRALI)] or overload [transfusion-associated cardiac overload (TACO)].

## Study strengths and limitations

Our study had several strengths. First, it was the first meta-analysis to specifically focus on ICU patients with sepsis. Second, even if the main part of our analysis consisted of a meta-analysis of observational studies, a structured approach was used and heterogeneity of the results was explored rigorously. Furthermore, risk of bias was assessed thanks to an adaptation of the Newcastle–Ottawa quality assessment scale, an accepted tool for cohort studies. Then, the funnel plot did not show any study effects and was in favor of a good research strategy.

Our study has, however, several limitations. First, there is only one randomized controlled trial. Consequently, this review mostly relies on observational studies. Then, the heterogeneity could not be fully explored. Indeed, several important factors such as leukodepletion, transfusion thresholds or age of the packed RBC could not be included into the analyses because they were not sufficiently reported. Third, it was not possible to integrate into our meta-analysis the data extracted from the studies dealing with EGDT. Finally, the two questions raised into this study may be quite limited and perhaps a more general question such as “how should we treat anemia in critically ill septic patients: only according to Hb laboratory values or according to anemia tolerance?” could be more appropriate. In that context, tolerance of anemia and thus transfusion thresholds should be considered as time-dependent variables. Furthermore, other treatments including transfusion-saving strategies such as administration of iron or erythropoietin in ICU, but also other transfusion triggers than Hb level such as lactate, ST elevation, ScVO_2_ or microcirculation should be integrated into the various analyses.

As a conclusion, restrictive RBCT strategies were associated with neither benefit nor harm compared to liberal strategies, and RBCT did not impact mortality but the occurrence of nosocomial infection. Because of sparse data and limits of observational studies, additional studies should be achieved. First, in order to explore the early phase of septic shock, a restrictive strategy could be assessed with a RCT. Then, because of the limits of RCTs in the case of adverse events such as ischemic events, we believed that observational studies with newer statistical causal models would be less biased and could lead to more definitive conclusions about the deleterious effect of transfusion in different subgroups and for different outcomes. Other transfusion triggers than hemoglobin should be explored.

## Additional files

Additional file 1: Search strategy, Boolean algorithms an definition used into the study.

Additional file 2: Additional Tables and Figures.

## Abbreviation

AKI: acute kidney injury
ALI: acute lung injury
CI: confidence intervals
EGDT: early goal-directed therapy
HR: hazard ratio
ICU: intensive care unit
MOOSE: meta-analysis of observational studies in epidemiology
NI: nosocomial infection
OR: odds ratio
PICO: participants, interventions, comparisons and outcomes
PRISMA: preferred reporting items for systematic reviews and meta-analysis
RBCT: red blood cell transfusion
RCT: randomized controlled trials
RR: relative risk
TACO: transfused associated cardiac overload
TRIM: transfusion-related immune modulation
