# Supplementary material for: Impact of transfusion on patients with sepsis admitted in intensive care unit: a systematic review and meta-analysis
Source: Ann Intensive Care. 2017 Jan 4;7:5. doi: 10.1186/s13613-016-0226-5 (PMC5209327; doi:10.1186/s13613-016-0226-5)
Supplement: Supplementary file 1 — Additional file 1: Search strategy, Boolean algorithms an definition used into the study. [file 13613_2016_226_MOESM1_ESM.docx]

**IMPACT OF TRANSFUSION ON PATIENTS WITH SEPSIS ADMITTED IN INTENSIVE CARE UNIT: A SYSTEMATIC REVIEW AND META-ANALYSIS**

## Additional file 1: *Search strategy, Boolean algorithms*

**The randomized controlled trials**

- Medline: ((((((((((threshold) OR liberal) OR restrictive) OR protocol) OR trigger) OR indicator) OR strategy) OR management) OR program) OR trigger)) AND (((((((((((((blood transfusion) OR transfusions, blood) OR transfusion, blood) OR blood transfusions) OR red blood cell) OR transfusion, red blood cell) OR red blood cell transfusions) OR transfusions, red blood cell) OR red blood cell transfusion) OR transfusions, erythrocyte) OR transfusion, erythrocyte) OR erythrocyte transfusions) OR RBC)) AND ((sepsis) OR septic shock)) AND (((((((((intensive care) OR critically ill) OR illnesses, critical) OR illness, critical) OR critical illnesses) OR critically illness) OR critical care) OR care, critical)))) AND ((((((Randomized controlled trials) OR Randomized Clinical) OR Control clinical trial) OR Controlled Clinical Trials) OR Randomized Clinical Trials) OR Randomized Trials)
- Cochrane: red blood cell transfusion* AND (intensive care OR critical care)
- Clinical trial: (blood transfusion OR transfusions OR red blood cell OR transfusions, erythrocyte OR RBC) AND (sepsis OR septic shock) AND (intensive care OR critically ill OR critical illnesses OR critically illness OR critical care)
- Web of science: (red blood cell transfusion*) AND sepsis AND (critical care OR intensive care) AND ((Randomized controlled trial*) OR (Randomized Clinical) OR (Control clinical trial*) OR (Controlled Clinical Trials) OR (Randomized Clinical Trials) OR (Randomized Trials))

**The cohort studies**

- Medline: ((((((((((((((((((((((((((((cohort study) OR incidence studies) OR studies, cohort) OR study, cohort) OR concurrent studies) OR studies, concurrent) OR concurrent study) OR study, concurrent) OR historical cohort studies) OR studies, historical cohort) OR cohort studies, historical) OR cohort study, historical) OR historical cohort study) OR study, historical cohort) OR analysis, cohort) OR analysis, cohort) OR cohort analyses) OR cohort analysis) OR closed cohort studies) OR cohort studies, closed) OR closed cohort study) OR cohort study, closed) OR study, closed cohort) OR studies, closed cohort) OR incidence study) OR studies, incidence) OR study, incidence)) AND ((((((((((threshold) OR liberal) OR restrictive) OR protocol) OR trigger) OR indicator) OR strategy) OR management) OR program) OR trigger)) AND (((((((((((((blood transfusion) OR transfusions, blood) OR transfusion, blood) OR blood transfusions) OR red blood cell) OR transfusion, red blood cell) OR red blood cell transfusions) OR transfusions, red blood cell) OR red blood cell transfusion) OR transfusions, erythrocyte) OR transfusion, erythrocyte) OR erythrocyte transfusions) OR RBC)) AND ((sepsis) OR septic shock)) AND (((((((((intensive care) OR critically ill) OR illnesses, critical) OR illness, critical) OR critical illnesses) OR critically illness) OR critical care) OR care, critical)))))
- Cochrane: (Cohort OR “observational study”) AND red blood cell transfusion* AND sepsis
- Web of science: ((cohort study) OR (incidence studies) OR (studies, cohort) OR (study, cohort) OR (concurrent studies) OR (studies, concurrent) OR (concurrent study) OR (study, concurrent) OR (historical cohort studies) OR (studies, historical cohort) OR (cohort studies, historical) OR (cohort study, historical) OR (historical cohort study) OR (study, historical cohort) OR (analysis, cohort) OR (analysis, cohort) OR (cohort analyses) OR (cohort analysis) OR (closed cohort studies) OR (cohort studies, closed) OR (closed cohort study) OR (cohort study, closed) OR (study, closed cohort) OR (studies, closed cohort) OR (incidence study) OR (studies, incidence) OR (study, incidence)) AND ((blood transfusion) OR (transfusions, blood) OR (transfusion, blood) OR (blood transfusions) OR (red blood cell) OR (transfusion, red blood cell) OR (red blood cell transfusions) OR (transfusions, red blood cell) OR (red blood cell transfusion) OR (transfusions, erythrocyte) OR (transfusion, erythrocyte) OR (erythrocyte transfusions) OR (RBC)) AND ((sepsis) OR (septic shock)) AND ((intensive care) OR (critically ill) OR (illnesses, critical) OR (illness, critical) OR (critical illnesses) OR (critically illness) OR (critical care) OR (care, critical))
- Clinical trial: (Cohort OR “observational study”) AND transfusion AND sepsis
- Medline: (prospective OR retrospective OR cohort OR observational) AND ("respiratory distress syndrome" OR "acute lung injury" OR "respiratory failure" OR "respiratory distress") AND ("risk factors" OR "associated factor*" ) AND (transfusion OR "red blood cell") AND ("sepsis" OR "septic shock")
- Web of science: idem
