# Supplementary material for: Impact of transfusion on patients with sepsis admitted in intensive care unit: a systematic review and meta-analysis
Source: Ann Intensive Care. 2017 Jan 4;7:5. doi: 10.1186/s13613-016-0226-5 (PMC5209327; doi:10.1186/s13613-016-0226-5)
Supplement: Supplementary file 2 — Additional file 2: Additional Tables and Figures. [file 13613_2016_226_MOESM2_ESM.docx]

**IMPACT OF TRANSFUSION ON PATIENTS WITH SEPSIS ADMITTED IN INTENSIVE CARE UNIT: A SYSTEMATIC REVIEW AND META-ANALYSIS**

**Additional file 2: Definitions used into the study**

Different score of severity at admission were used to assess the probability of Death in intensive care unit. Thus, we used the APACHE 2 Score[1], the APACHE 3 score[2], the SAPSS 2 score[3] and the SOFA score[4,5].

Table S1: Patients characteristics and primary mortality outcome of the included randomized controlled trial

| **Authors** | **Included patients and intervention** | **Outcomes** | **RBCT in restrictive group** | **Others main results** |
| --- | --- | --- | --- | --- |
| **Holst**  [6] | 998 septic shock patients and Hb <9g/dL  • Liberal group: Hb over 9g/dL : n = 496;  • Restrictive group: Hb between 7 and 9g/dL: n =502; | Restrictive/liberal  90-day mortality rate: 43% vs 45%,p=0.44  RR 0.94[0.78-1.09], p=0.44 | Restrictive: 1545 transfusions; 1 unit[0-3] and 176(36.1%) without any transfusion;  Liberal: 3088 transfusions, 4[2-7] and 6 (1.2%) without any transfusion | Protocol temporarily excluded*:  Restrictive: 29/488 (5.9%) vs liberal: 11/489(2.2%)(p=0.004).  Ischemic event during ICU stay:  Restrictive: 7.2% vs liberal: 8%.  Lowest median Hemoglobin after day 2: restrictive: 7.6 g/dL; liberal: 9.3 g/dL. |
| RBCT: Red Blood Cell Transfusion; Hb: Hemoglobin; OR: Odd Ratio; RR: Relative Risk;  *exclusion for myocardial ischemia or other ischemia, life threatening bleeding or ECMO therapy (extracorporeal membrane oxygenation). | | | | |

Table S2: Quality assessment of the randomized controlled trials included into the systematic review. (Hb: Hemoglobin);

| **Source** | **Random sequence**  **generation**  **(selection bias)** | **Allocation concealment**  **(selection bias)** | **Blinding**  **(performance bias and detection bias)** | **Withdrawals Incomplete outcome data**  **(attrition bias)** | **Protocol Violations** | **Selective reporting**  **(reporting bias)** |
| --- | --- | --- | --- | --- | --- | --- |
| **Holst**  [6] | **Low risk**  🡪 computer-generated | **Low risk**  🡪 centralized, web-based | **Unclear**  No blinding, pragmatic trial | **Low risk**  Lower Hb threshold 🡪 38 withdrawals  Higher Hb threshold 🡪 24 withdrawals | A total of 29 of 488 patients (5.9%) in the lower threshold group and 11 of 489 (2.2%) in the higher threshold group had the protocol temporarily  suspended (P = 0.004) | **Low risk** |

Table S3: Characteristics of the cohort studies included into the systematic review;

| **Author** | **Type of study** | **Inclusion criteria** | **Frequency**  **of septic**  **patients** | **Outcome** | **Statistical model** | **Population**  **size** | **main results** | **confounding factors** |
| --- | --- | --- | --- | --- | --- | --- | --- | --- |
| **Micek**  **2005**[7] | Prospective  Monocentric  01/2002 to 04/2004 | septic shock  and treatment  with dotrecogin  alpha | 100 | H death | Multi LR | 102 | In-hospital mortality rate (42.2%)  Mean(sd)(dead/alive)  # units of RBC transfused  1.7 ± 2.5 vs 1.3 ± 2.7;p=0.043  NS in multivariate analysis |  |
| **Iscimen**  **2008**[8] | Prospective  Monocentric  03/2004 to 04/2007 | septic shock | 100 | ICU and H death | Multi LR | 71(ALI)  +91(no ALI) | OR: 2.75[1.22-6.37];p=0.016  **Outcome**  ICU Death ,n(%)ALI:27 (38),no ALI 10 (11), p = 0.001  H Death , n(%)ALI:36 (51),no ALI 16 (18) , p< 0.001 | delayed GDT, delayed AB, RR, chemotherapy, chronic OH, transfusion, aspiration, diabetes mellitus |
| **Fuller 2010**[9] | Retrospective  Monocentric  02/2005 to 06/2008 | Septic shock | 100 | H death | Uni LR | 93  (34 transfused) | N(%)(transfused/not transfused)  In-hospital mortality rate: 14 (41.2%);20(33.9%); p<0.05  ILOS (days) 11.4;3.8;p<0.05 HLOS (days) 25.9;12.5;p<0.05 |  |
| **Juffermans**  **2010**[10] | Retropective  Multicentric  01/2004 to 11/2007  NICEdatabase | septic patient | 100 | NI | Multi LR | 134  (67 transfused) | OR: 1.25[1.04-1.51],p=0.02 | immunosuppressive medication, Acute Physiology and Chronic Health Evaluation II score, malignancy, HIV infection, alcohol abuse, or diabetes mellitus |
| **Parson**  **2011**[11] | Prospective  Multicentric  2000 to 2005  ARDS Network FACTT study | ALI and sepsis | 100 | death at D28  and D90 | Multi LR | 285  (53 transfused) | D28: transfused: 10(50%), no transfused: 19(29%)  D28: OR=1.49[ 0.77 -2.90]; P = 0.23  D90: aOR = 1.55[0.81- 2.96]; p=0.19 | AGE, sex, APACHE III score, FACTT randomized arm as cofactors. |
| **Plataki**  **2011**[12] | Prospective  Monocentric  07/2005 to 09/2007 | septic shock | 100 | AKI (RIFLE) | Multi LR | 390 | OR: 5.22 [2.1 to 15.8], p= 0.001  In-hospital mortality rate , n (%) 52 (34)vs 115 (49) ;p=0.005 | Baseline GFR, smoking history, BMI, Use of ACEI or ARB, intra-abdominal sepsis, APACHE III, adequate resuscitation, time to adequate AB |
| **Perner**  **2012**[13] | Prospective  Monocentric  02/2009-06/2009 | septic shock | 100 | death D90 | LR | 164  (99 transfused) | OR: 1.1 [0.5–2.5]  D30 : 40% | surgery/SAPS II admission/  SOFA score day 1 |
| **Park**  **2012**[14] | Prospective  Multicentric  05/2005 to 02/2009 | severe sepsis  or septic shock | 100 | death D7,  H death | cox model/  PS  matching | 1054  (407 transfused)  152 pairs | All cohort; multi cox:  RBCT:HR[IC 95%]  D7:0.42[0.19-0.5];p=0.026  D28:0.43[0.29-0.62];p<0.001  In-hospital mortality rate :0.51[0.39-0.69];p<0.001;  Propensity match cohort: (%)(transfu/ no transfu)  D7: 9.2% vs. 27.0%; p < .001,  D28: 24.3% vs. 38.8%; p = 0.007  In-hospital mortality rate : 31.6% vs. 42.8%; p =0.044  HLOS(days): 23[IQR 11–39] vs 13[IQR 5–25]; p<0.001  HR:0.35[0.23–0.53],p<0.001 | age, sex, comorbid diseases, source of infection, Sequential Organ Failure Assessment and APACHE II scores at admission, Hb concentration at admission, type of organ dysfunction, mechanical ventilation, continuous renal replacement therapy, and RBC transfusion |
| **Rosland**  **2014**[15] | Prospective  Multicentric  5 month period | septic shock | 100 | death at D90 | Multi LR | 213  (95 transfused ) | OR: 1.72 (0.91-3.24);p=0.1  28-day mortality rate, all 102 (48) transfused 56 (59),no transfused 46 (39),p= 0.004  90-day mortality rate: 63 (66) vs 51 (43), p= 0.0008 | medical or surgical origin, SAPSS II , SOFA at D1, and covariates with p val <0.1 |
| **Sadaka**  **2014**[16] | prospective DB  monocentric  06/2011  to 03/2013 |  | 100 | H Death | Hospital death  matching | 396 | RBC 41%(N=46) vs. No RBC 39.4%(N=71)  OR: 0.8[ 0.4 - 1.7], p = 0.6 | matching: age, illness severity, bundle |
| **Na**  **2012**[17] | Prospective Multicentric  07/2008 to 12/2009 | severe sepsis  or septic shock. | 100 |  | Hospital death  Multi LR | 556 | In-hospital mortality rate:  transfusion: OR: 1.41 [0.85, 2.35]] |  |
| **Erbay**  **2006**[18] | Retrospective  Monocentric  01/1998 to 02/2002  electronic DB | ICU and  catheter initially  infected | 100 | catheter NI | cox model | 73 | (recurrent NI/no recurrent NI)(N(%))  blood administration: 17(68); 30(63) ;p=0.049  HR : 2.3[1.02—5.67], p = 0.049 | gender, age, patient unit (ICU,  Burns Unit, or elsewhere in the hospital), multilumen  catheters, infusate (blood or TPN), and reinsertion site  (guidewire vs. different site). |
| ALI : Acute lung injury ; AKI : Acute Kidney Injury ; ARDS : Acute Respiratory Distress Syndrom ; D : Day ; ICU : Intensive care unit ; H : hospital ; LR : Logistic regression ; MV : Mechanical Ventilation ; NI : nosocomial Infection ; PS : propensity score ; RBC : Red Blood Cell ; RR : relative risk ; Ventilator Associated Pneumonia; NS: Non Significant; OR: Odd Ratios; GDT: Goal Directed Therapy; AB: Antibiotherapy; RR: Relative Risk; ILOS: ICU Length Of Stay; PS: Propensity Score; WBC: White Blood Cells. | | | | | | | | |

Table S4: Quality assessment of the cohort studies included into the systematic review

| **Authors** | **Representativity of the Intervention Cohort** | **Ascertainment Cohort (computerized versus manual**  **control of the data)** | **Outcome of interest**  **not present on admission** | **Confounding**  **Factors** | **Assessment of utcome**  **(blindness, digital or manual records)** | **Missing data** |
| --- | --- | --- | --- | --- | --- | --- |
| **Micek**  **2005** [7] | **Low risk**  All patients treated with drotrecogin alfa (activated)  for severe sepsis were eligible | **Low risk**  recorded prospectively, prospective, observational cohort study, recorded relevant data from the medical records, bedside nursing charts, and the hospital’s mainframe computer for reports of microbiologic studies (Gram’s stains and cultures of blood, urine, sputum, lower respiratory tract specimens, tissue, and wounds). All pharmacotherapies administered in the emergency department, general medical ward, and intensive care unit were evaluated by using patients’ medical records and the hospital’s computerized bedside workstations | **Low risk**  YES | **High Risk**  NO | **Low risk**  Record linkage,  prospective record | **Low risk**  NO |
| **Iscimen**  **2008** [8] | **Low risk**  Selected group  (septic shock without ALI) | **Low risk**  Daily screening identified consecutive  adults | **High Risk**  ( not clear for the achievement of double assessments and missing data) | **Low risk**  YES | **Unclear**  Record (P/F chest X ray) | **High Risk**  YES(P/F(PaO2/FIO2 (IQR) n = 112) for 160 patients |
| **Fuller**  **2010**[9] | **Low risk**  Septic shock patients | **Low risk**  Retrospective but prospective record Surviving Sepsis Campaign Chart Review database and linked to Project IMPACT database | **Low risk**  YES | **High Risk**  NO | **Low risk**  Two abstractors (MG and CS). CS has had extensive experience and training in database management and chart review Regular meetings and monitoring of data  collection were performed and the chart reviewers were  blinded to study hypothesis | **Unclear**  we cannot exclude the  possibility of missing data, which may  cause undetected differences in baseline characteristics. For example, the PRBC group received higher volumes no |
| **Juffermans**  **2010** [10] | **Low risk**  Sepsis | **Low risk**  Retrospective (Patients with sepsis were retrieved from the National Intensive Care Evaluation (NICE) minimal data set) patient digital medical system, other NICE data set, blood transfusion service computer service. Data on… were retrieved from the patient digital medical system. Transfusion data were extracted from the hospital blood transfusion service computer system, | **Low risk**  YES | **Low risk**  YES | **High Risk**  Retrospective record and only bacterial infections were studied | **Unclear** |
| **Parson**  **2011** [11] | **Low risk**  FACTT enrolled 1,000 subjects  within 48H of a new ALI diagnosis  identified subjects with sepsis and shock within  the FACTT database | **Low risk**  a secondary analysis of the Acute Respiratory Distress Syndrome Network (ARDSNet) FACTT, a multicenter, randomized, controlled trial | **Low risk**  YES | **Low risk**  YES | **Low risk**  All required data  elements for our secondary analysis were available in  their entirety from the FACTT database | **High Risk**  YES+++  performed multiple imputation by chained  equations to account for missing data. We excluded 328 subjects (33%) with an  ALI risk factor of trauma (18 subjects), multiple transfusion (6 subjects) or missing transfusion data (304 subjects). Of these 285 subjects, 85 (30%) met all transfusion indicators outlined above. |
| **Plataki**  **2011** [12] | **Low risk**  Septic shock | **Low risk**  a retrospective analysis of a prospectively collected cohort of consecutive adults. Study investigators reviewed monitoring logs capturing respiratory rate, heart rate, arterial and central venous pressures (CVP), UO, laboratory findings, infusions, and other treatments | **Low risk**  those with ESKD on chronic renal replacement therapy (RRT), patients in whom care was withdrawn within 6 hours of onset of septic shock, those with pre-existing AKI at the onset of shock, those in whom the time of onset of shock started before hospital admission or could not be accurately determined, and readmissions were excluded | **Low risk**  YES | **Low risk**  record | **Low risk**  NO |
| **Perner**  **2012** [13] | **Low risk**  Septic shock | **Low risk**  prospective, cohort study  All data were collected on paper case report forms  by a single investigator (SS) and entered into an  Excel data sheet. | **Low risk**  YES | **Low risk**  YES | **Low risk**  30-day and 90-day mortality rate  from the National Patient Registry. | **Low risk**  NO |
| **Park**  **2012**[14] | **Low risk**  Septic shock | **Low risk**  Prospective, multicenter obser­vational study Prospective informatics DB. We analyzed the database from the Korean Sepsis Registry System, with the aim of evaluating the clinical characteristics and outcome of severe sepsis in Korean patients. | **Low risk**  YES | **Low risk**  YES | **Low risk**  Digital record through the Korean Sepsis Registry System. Patients A coordinating center (Korea University Anam Hospital) supported data registration at each center, and all data were checked to be within acceptable ranges. | **Low risk**  NO |
| **Rosland**  **2014** [15] | **Low risk**  Septic shock | **Low risk**  prospective cohort study of RBC transfusion  Data were recorded for Data were registered  on paper case report forms at the respective ICUs and merged into the study database by two investigators. | **Low risk**  YES | **Low risk**  YES | **Low risk**  The latter by the use of the civil personal registration number in the administrative system for Danish hospitals (GS Open). | **Low risk**  Presented were complete case analyses. All patients were included in the multivariate analysis and we used the obtained SAPS II and SOFA score. Thus, we did not impute missing score components. |
| **Sadaka**  **2014**[16] | **Low risk**  Septic shock | **Low risk**  Data are prospectively collected; This study is a retrospective review of this database. Prospective database but retrospective analysis | **Low risk**  YES | **Low risk**  YES | **Low risk**  Hospital mortality | **Unclear**  ? |
| **Erbay**  **2006** [18] | **Low risk**  Catheter infected patients in ICU | **Low risk**  retrospective cohort study of catheters Electronic DB  Data were extracted to a  structured electronic database. | No, **Unclear** if it is the first…. All the patients who had a CR-BSI between January 1998  February 2002 were | **Low risk**  YES |  | **Unclear** |
| ALI: Acute Lung Injury; P/F: PaO2 over FiO2 ratio; UO: Urinary Output; ICU: Intensive Care Unit; MV: Mechanical Ventilation; ARDS: Acute respiratory Distress Syndrom; SICU: Surgical ICU. | | | | | | |


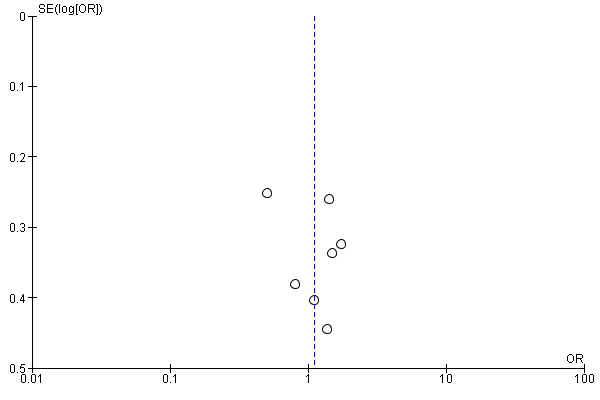


Figure S1: Funnel plot of the cohort studies dealing with mortality rate


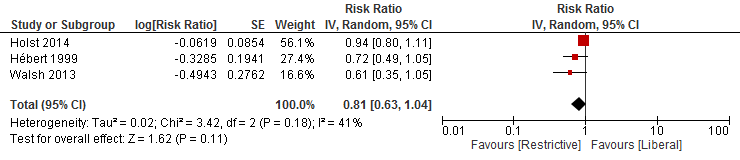


Figure S2: Forest Plot of Risk Ratio: Impact of a restrictive versus a liberal transfusion strategy on the mortality rate; CI: Confidence Interval, IV: inverse variance, SE: standard error.

1. Knaus WA, Draper EA, Wagner DP, Zimmerman JE. APACHE II: a severity of disease classification system. Crit. Care Med. 1985;13:818–29.

2. Knaus WA, Wagner DP, Draper EA, Zimmerman JE, Bergner M, Bastos PG, et al. The APACHE III prognostic system. Risk prediction of hospital mortality for critically ill hospitalized adults. Chest. 1991;100:1619–36.

3. Le Gall JR, Lemeshow S, Saulnier F. A new Simplified Acute Physiology Score (SAPS II) based on a European/North American multicenter study. JAMA. 1993;270:2957–63.

4. Vincent JL, de Mendonça A, Cantraine F, Moreno R, Takala J, Suter PM, et al. Use of the SOFA score to assess the incidence of organ dysfunction/failure in intensive care units: results of a multicenter, prospective study. Working group on “sepsis-related problems” of the European Society of Intensive Care Medicine. Crit. Care Med. 1998;26:1793–800.

5. Ferreira FL, Bota DP, Bross A, Mélot C, Vincent JL. Serial evaluation of the SOFA score to predict outcome in critically ill patients. JAMA J. Am. Med. Assoc. 2001;286:1754–8.

6. Holst LB, Haase N, Wetterslev J, Wernerman J, Guttormsen AB, Karlsson S, et al. Lower versus Higher Hemoglobin Threshold for Transfusion in Septic Shock. N. Engl. J. Med. 2014;371:1381–91.

7. Micek ST, Isakow W, Shannon W, Kollef MH. Predictors of hospital mortality for patients with severe sepsis treated with drotrecogin alfa (activated). Pharmacotherapy. 2005;25:26–34.

8. Iscimen R, Cartin-Ceba R, Yilmaz M, Khan H, Hubmayr RD, Afessa B, et al. Risk factors for the development of acute lung injury in patients with septic shock: an observational cohort study. Crit. Care Med. 2008;36:1518–22.

9. Fuller BM, Gajera M, Schorr C, Gerber D, Dellinger RP, Parrillo J, et al. The impact of packed red blood cell transfusion on clinical outcomes in patients with septic shock treated with early goal directed therapy. Indian J. Crit. Care Med. Peer-Rev. Off. Publ. Indian Soc. Crit. Care Med. 2010;14:165–9.

10. Juffermans NP, Prins DJ, Vlaar APJ, Nieuwland R, Binnekade JM. Transfusion-Related Risk of Secondary Bacterial Infections in Sepsis Patients: A Retrospective Cohort Study. Shock. 2011;35:355–9.

11. Parsons EC, Hough CL, Seymour CW, Cooke CR, Rubenfeld GD, Watkins TR. Red blood cell transfusion and outcomes in patients with acute lung injury, sepsis and shock. Crit. Care. 2011;15:R221.

12. Plataki M, Kashani K, Cabello-Garza J, Maldonado F, Kashyap R, Kor DJ, et al. Predictors of acute kidney injury in septic shock patients: an observational cohort study. Clin. J. Am. Soc. Nephrol. CJASN. 2011;6:1744–51.

13. Perner A, Smith SH, Carlsen S, Holst LB. Red blood cell transfusion during septic shock in the ICU. Acta Anaesthesiol. Scand. 2012;56:718–23.

14. Park DW, Chun B-C, Kwon S-S, Yoon YK, Choi WS, Sohn JW, et al. Red blood cell transfusions are associated with lower mortality in patients with severe sepsis and septic shock: a propensity-matched analysis*. Crit. Care Med. 2012;40:3140–5.

15. Rosland RG, Hagen MU, Haase N, Holst LB, Plambech M, Madsen KR, et al. Red blood cell transfusion in septic shock - clinical characteristics and outcome of unselected patients in a prospective, multicentre cohort. Scand. J. Trauma Resusc. Emerg. Med. 2014;22:14.

16. Sadaka F, Trottier S, Tannehill D, Donnelly PL, Griffin MT, Bunaye Z, et al. Transfusion of red blood cells is associated with improved central venous oxygen saturation but not mortality in septic shock patients. J. Clin. Med. Res. 2014;6:422–8.

17. Na S, Kuan WS, Mahadevan M, Li C-H, Shrikhande P, Ray S, et al. Implementation of early goal-directed therapy and the surviving sepsis campaign resuscitation bundle in Asia. Int. J. Qual. Health Care J. Int. Soc. Qual. Health Care ISQua. 2012;24:452–62.

18. Erbay A, Ergönül Ö, Stoddard GJ, Samore MH. Recurrent catheter-related bloodstream infections: risk factors and outcome. Int. J. Infect. Dis. 2006;10:396–400.
